# Semi-Annual Meeting of the Medical Society of the State of New York

**Published:** 1901-11

**Authors:** 


### Semi-Annual Meeting of the Medical Society of the State of New York.

IN THE number of the JOURNAL for May, 1851, appears the notice of a semi-annual meeting of the State Medical Society, to be held in Buffalo the second Tuesday in June, with the request that "as it is a new movement on the part of the society it should be responded to by a full meeting." The meeting was a success, but for some reason, the plan was not continued. At the annual meeting of the society, held at Albany last January, just fifty years later, again it was decided to hold a semi-annual meeting—this time at the Academy of Medicine in New York City, October 15 and 16, 1901—and this like its predecessor of 1851, was a pronounced success. The meeting was as well attended as any ever held in the Academy and the high standard of the papers read, as well as the scientific ability of the readers made a most favorable impression upon the hundreds who sought and obtained admission to the sessions.

On Tuesday evening, October 15, a symposium on Some of the Diseases of the Liver and Bile Passages was given, which was participated in by Drs. Charles G. Stockton, of Buffalo; Richard C. Cabot and S. J. Mixter, of Boston; Simon Flexner, of Philadelphia; L. S. Pilcher, of Brooklyn; C. L. Gibson, F. Tilden Brown and B. F. Curtis, of New York.

On Wednesday evening the Medical Society of the County of New York tendered a reception to the members and guests which proved a most enjoyable entertainment.

Among some of the special features of the meeting were the demonstration of the vascular supply of the kidney, by Dr. Howard Kelly, of Baltimore; a discussion on the treatment of croupous pneumonia, led by Dr. J. C. Wilson, of Philadelphia; the report of the physicians on President McKinley's case, read by Dr. M. D. Mann, of Buffalo, supplemented by impromptu remarks by Dr. Herman Mynter, of Buffalo.

Forty out of the fifty papers on the program, besides the symposium, were read and discussed, and the verdict rendered was that no better meeting of the state society had ever been held. Even the annual meetings at Albany rarely equal and never excel it in quality.

Although medical society meetings are frequent in New York State, there is yet room for a semi-annual meeting of the state society and the innovation, again started, might well be followed in succeeding years.

Much credit for the success of the meeting is due to the President, Dr. Henry L. Elsner, of Syracuse, ably assisted by the business committee of which Dr. Nathan Jacobson, of Syracuse, is chairman.

